# EGFR and mTOR as therapeutic targets in glioblastoma

**DOI:** 10.18632/oncotarget.27094

**Published:** 2019-07-30

**Authors:** Michael W. Ronellenfitsch, Anna-Luisa Luger, Joachim P. Steinbach

**Affiliations:** Dr. Senckenberg Institute of Neurooncology, University Hospital Frankfurt, Goethe University, Frankfurt am Main, Germany; University Cancer Center Frankfurt, University Hospital Frankfurt, Goethe University, Frankfurt am Main, Germany; German Cancer Consortium, Partner Site Frankfurt/Mainz, Frankfurt am Main, Germany; Frankfurt Cancer Institute, University Hospital Frankfurt, Goethe University, Frankfurt am Main, Germany

**Keywords:** glioblastoma, EGFR, mTOR, DDIT4, tumor microenvironment

**Figure 1:**
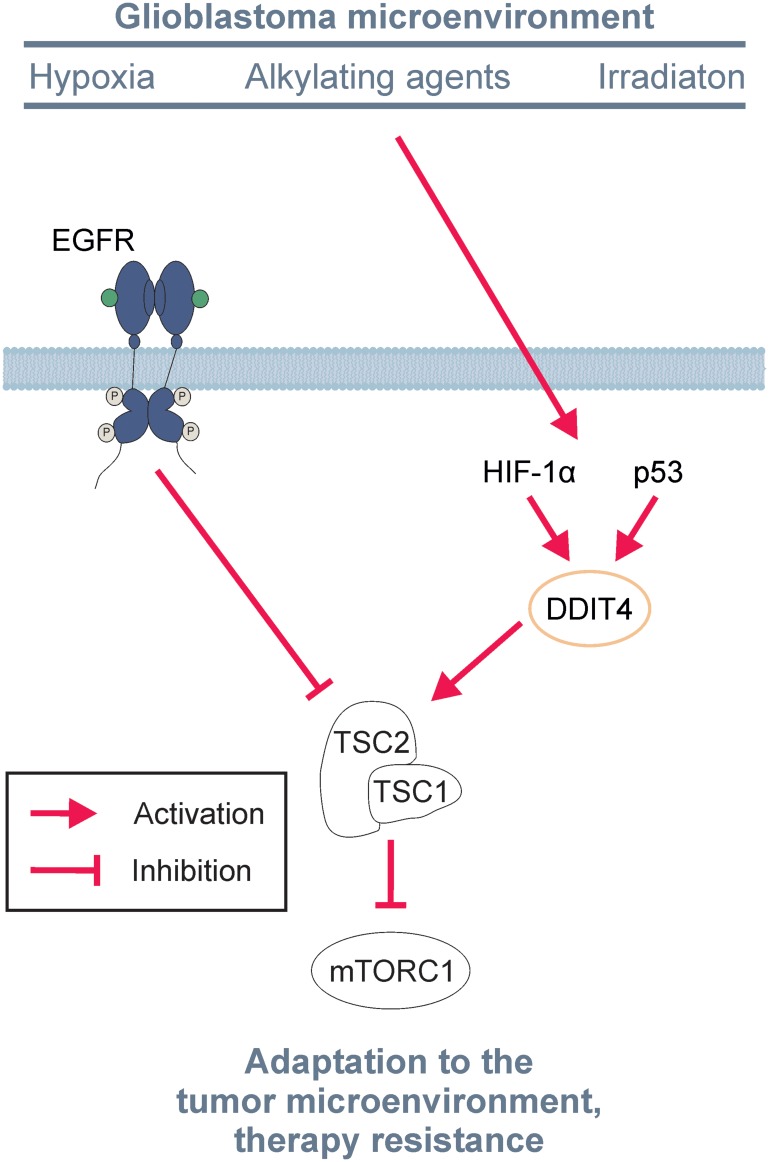
Scheme of EGFR signaling and DDIT4-mediated adaptive processes. Conditions of the glioblastoma microenvironment including hypoxia, alkylating therapy or irradiation trigger induction of DDIT4 which activates TSC1/2 to inhibit mTORC1 and can counteract epidermal growth factor receptor (EGFR)-mediated TSC1/2 inhibition. Inhibition of mTORC1 ultimately induces adaptive processes to cope with external stressors.

The quest for new and improved therapies for glioblastoma (GB) has been mostly unsuccessful in more than a decade despite significant efforts. The few exceptions include the optimization of classical alkylating chemotherapy by including lomustine in the first line regimen for GB with a methylated MGMT promoter and tumor treating fields [1, 2]. The GB signaling network has been well-characterized and genetic alterations resulting in activation of receptor tyrosine kinases and especially epidermal growth factor receptor (EGFR) and downstream mammalian target of rapamycin complex 1 (mTORC1) signaling were found in the majority of GBs [3]. Therefore, many hopes have rested on targeted therapies. However, the results from clinical trials have been largely disappointing [4]. Nevertheless, unplanned retrospective subgroup analyses of the patient cohorts of negative clinical trials indicated that dysregulation or activation of signaling could be a predictive factor for susceptibility to pathway inhibition: Tumors with enhanced levels of mTORC1 activation markers, including phosphorylated ribosomal protein S6 and phosphorylated mTOR itself, appeared to respond to pathway inhibition by the EGFR antibody nimotuzumab or the mTORC1 inhibitor temsirolimus [5, 6]. In contrast, the perils of mTORC1 inhibitor treatment in unselected GB patient cohorts with a “one size fits all approach” have been demonstrated by the recently published RTOG 0913 trial, where mTOR inhibition had detrimental effects with reduced overall survival [7]. While, except for a theoretical compensatory Akt pathway activation or potential toxicity related under-treatment, no plausible mechanistic explanations have been offered, we could previously demonstrate that EGFR and mTORC1 are at the center of GB cell adaptive responses (Figure 1). Conditions of the tumor microenvironment including and nutrient deprivation cause downregulation of mTORC1 signaling for metabolic adaptation (Figure 1) [8]. Accordingly, inhibition of mTORC1 or EGFR confer protection against hypoxia-induced cell death while constitutive activation of mTORC1 renders GB cells susceptible to hypoxia-induced cell death [8–10]. Thus, the activity of the EGFR-mTORC1 axis is pivotal for survival under starvation conditions, but on the other hand it also promotes the neoplastic phenotype of GB cells. How then do GB cells manage to find appropriate degrees of signaling for their respective condition? Physiologically, GB cells can fine-tune signaling on the basis of microenvironmental conditions via the protein DNA damage-inducible transcript 4 (DDIT4) which is a physiological inhibitor of mTORC1 signaling (Figure 1) [11]. DDIT4 is regulated on a transcriptional level by several factors including p53 and HIF-1α. In line, both hypoxia as well as DNA stress by alkylating chemotherapy or radiotherapy induced DDIT4 in some GB cell lines. The relevance of this adaptive mechanism is underscored by genetic experiments demonstrating an increased sensitivity when DDIT4 gene expression is suppressed and reduced sensitivity when DDIT4 gene expression is induced to the various cell stressors depending on the cell line [11]. These results align with the finding that mTORC1 inhibitors can exert protective effects and it is only plausible that a pre-emptive adjustment of signal transduction by inhibitor treatment or genetic DDIT4 induction prepares GB cells for the following challenge [8, 11]. Therefore, DDIT4 could be a candidate for therapeutic inhibition to better treat GB.

What consequences do these findings have for clinical traials with inhibitors of EGFR or mTOR signal transduction? To fully exploit the potential of inhibitors, biomarkers have to be developed to identify tumors with defective wiring of signal transduction. Additionally, these findings may influence the potential armamentarium for combinatorial drug treatment. A direct combination with drugs that cause local hypoxia or nutrient deprivation like antiangiogenic drugs might result in reciprocal annulations of effects. Depending on the half life and pharmacokinetics of the drugs, stepwise treatment algorithms could be an option to prevent antagonistic effects. While glioma-associated microglia/macrophages (GAMs) can make up for more than half of the cells of a GB tumor, the role of signal transduction inhibitors on GAMs has only marginally been addressed but may nevertheless influence treatment efficacy. It is interesting to note that GBs with more extensive microglia infiltration appeared more responsive to EGFR inhibition with the EGFR antibody nimotuzumab [6]. This may be but one of several exciting connections between microenvironment, tumor and immune cells that await discovery and exploitation for therapeutic purposes.

These considerations give reason for cautious optimism that, while the path to clinical development of EGFR and mTORC1 inhibitors for GB treatment has certainly been strenuous, the advancement of understanding of the wiring of GB signal transduction will eventually reveal actionable targets at least in some tumor and patient subgroups.
